# Differential Effects of Dietary Fat Content and Protein Source on Bone Phenotype and Fatty Acid Oxidation in Female C57Bl/6 Mice

**DOI:** 10.1371/journal.pone.0163234

**Published:** 2016-10-03

**Authors:** Emily A. Sawin, Bridget M. Stroup, Sangita G. Murali, Lucas M. O’Neill, James M. Ntambi, Denise M. Ney

**Affiliations:** 1 Department of Nutritional Sciences, University of Wisconsin-Madison, Madison, Wisconsin, United States of America; 2 Department of Biochemistry, University of Wisconsin-Madison, Madison, Wisconsin, United States of America; State University of Rio de Janeiro, BRAZIL

## Abstract

**Background:**

Glycomacropeptide (GMP) is a 64-amino acid glycophosphopeptide released from κ-casein during cheesemaking that promotes satiety, reduces body fat, increases bone mass and infers prebiotic and anti-inflammatory effects. The impact of adiposity and gender on bone health is unclear.

**Objective:**

To determine how feeding female mice diets providing 60% Fat Kcal (high-fat) or 13% Fat Kcal (control) with either GMP or casein as the protein source impacts: body composition, ex vivo fatty acid oxidation, bone (femoral) biomechanical performance, and the relationship between body composition and bone.

**Methods:**

Weanling female C57Bl/6 mice were fed high-fat (60% Fat Kcal) or control diets (13% Fat Kcal) with GMP or casein from 3 to 32 weeks of age with assessment of body weight and food intake. Body composition was assessed by dual-energy X-ray absorptiometry (DXA). Fatty acid oxidation was measured in liver, muscle, and fat tissues using ^14^C-palmitate. Plasma concentrations of hormones and cytokines were determined. Bone biomechanical performance was assessed by the 3-point bending test.

**Results:**

Female mice fed high-fat diets showed increased fatty acid oxidation capacity in both gastrocnemius muscle and brown adipose tissue compared to mice fed the control diets with a lower fat content. Despite increased fat mass in mice fed the high-fat diets, there was little evidence of glucose impairment or inflammation. Mice fed the high-fat diets had significantly greater total body bone mineral density (BMD), femoral BMD, and femoral cross-sectional area than mice fed the control diets. Femora of mice fed the high-fat diets had increased yield load and maximum load before fracture, consistent with greater bone strength, but reduced post-yield displacement or ductility, consistent with bone brittleness. Female mice fed a high-fat GMP diet displayed increased fat oxidation capacity in subcutaneous fat relative to mice fed the high-fat casein diet. Regardless of dietary fat content, GMP increased total body bone mineral content and femur length. The prebiotic properties of GMP may mediate the beneficial effects of GMP on bone.

**Conclusions:**

Female mice adapt to high-fat feeding by increasing oxidative capacity in muscle tissue and to a lesser extent brown adipose tissue. High-fat feeding in female mice leads to development of a bone phenotype where femora show increased BMD and are stronger, yet more brittle. The increased brittleness of bone was associated with increased body fat content due to high-fat feeding. In summary, high-fat feeding in female mice increases mineralization of bone, but negatively impacts bone quality resulting in brittle bones.

## Introduction

Glycomacropeptide (GMP), also known as caseinomacropeptide, is a bioactive 64-amino acid glycophosphopeptide isolated from the C-terminal end of κ-casein in bovine milk during the manufacture of cheese [1]. GMP is released into the whey and comprises about 20–25% of proteins in whey protein isolate and whey protein concentrate. GMP has a unique amino acid profile in that in its pure form, it is completely devoid of the aromatic amino acids (phenylalanine, tyrosine, and tryptophan); thus, GMP provides a source of low-phenylalanine protein for those individuals diagnosed with phenylketonuria (PKU) [2]. GMP also contains two- to three-fold greater concentrations of isoleucine and threonine, respectively, compared to typical dietary proteins [3]. It is at these threonine residues that GMP is glycosylated by mucin-type carbohydrate residues [4]. These carbohydrate residues and unique amino acid composition contribute to the prebiotic properties of GMP [5].

GMP demonstrates a number of interesting biological activities including anti-inflammatory effects in rat models of colitis and ileitis [6, 7] as well as the potential to promote satiety in humans [8–11]. One proposed mechanism through which GMP may act to promote satiety is through modulation of gastrointestinal hormones including cholecystokinin (CCK) [12, 13] and ghrelin [10]. The literature regarding GMP’s ability to limit food intake in human subjects is mixed, as some studies report reduced food intake after consuming a GMP preload meal and others show no difference when compared to other protein fractions of whey [8, 11, 14, 15]. In rodent models, GMP also demonstrates anti-obesity properties and was shown to reduce fat mass in Wistar rats [16] and in PKU mice [17]. Female mice fed the GMP diet had significantly lower fat mass than female mice fed the casein diet and a significantly lower respiratory exchange ratio consistent with increased fat oxidation. Male mice did not show the same significant effect [17]. Male Sprague-Dawley rats fed a high-fat diet supplemented with GMP showed decreased body weight gain, adipocyte size, and plasma triglyceride concentration suggesting that GMP may improve fat catabolism in the liver and adipose tissue [18]. Gaps in the literature exist regarding the mechanisms by which GMP acts to reduce adiposity and increase satiety, and its differential effects in males and females.

Additional beneficial biological activities of GMP include promoting bone and dental health. Administration of GMP exerts inhibitory activity against enamel demineralization and promotes tooth enamel remineralization [19] as well as increases calcium bioavailability to inhibit bone loss in ovariectomized rats [20]. Administration of GMP to mice fed a low-calcium diet improves calcium content in the femur, consistent with increased calcium bioavailability [21]. In the PKU mouse model, the amino acid diet exacerbated skeletal fragility associated with the PKU genotype, but the GMP diet was able to significantly improve this phenotype and increase the maximum load a femur can withstand before fracture suggesting its ability to improve bone biomechanical performance [22].

Multiple investigators have examined the relationship between adiposity and bone mineral density (BMD) as well as fracture risk. In the past, obesity has been associated with a protective effect on bones as the heavier weight increases the load on the bone leading to increased BMD [23]. More recently, obesity is being associated with a higher risk of fracture [24]. Interestingly, subcutaneous fat has a positive correlation with increased bone mass whereas visceral fat, which is deposited in greater amounts in human males [25], is deleterious to bone [26]. Gender and menopausal status also affect how fat mass correlates with bone health [27]. In male C57Bl/6 mice, a high-fat diet increases size, or cross-sectional area, of the bone; however, the biomechanical properties of the bone are decreased [28]. A comprehensive assessment of the effect of a high-fat diet on the biomechanical properties of bone in female mice has not been reported. Future work is needed to determine how adiposity and gender impact fracture risk, which is affected by both bone quantity and bone quality.

Our objective was to investigate how feeding female mice diets providing 60% Fat Kcal (high-fat) or 13% Fat Kcal (control) with either GMP or casein as the protein source impacts: body composition, ex vivo fatty acid oxidation, bone (femoral) biomechanical performance, and the relationship between body composition and bone. We tested the hypotheses that: 1) high-fat feeding increases fatty acid oxidative capacity, but has a negative impact on the bone phenotype and 2) providing GMP as the primary source of dietary protein improves body composition and the bone phenotype compared with casein.

## Materials and Methods

### Animals and Experimental Design

The University of Wisconsin-Madison Institutional Animal Care and Use Committee approved the animal care protocol, number A005125, used in this study. Weanling female C57Bl/6 mice were purchased from Jackson Laboratory (Bar Harbor, ME) and singly housed in shoe-box cages. The experimental design included 4 treatment groups in a 2x2 factorial design, which included 2 main effects and their interaction: fat concentration of diet, 60% fat Kcal or 13% fat Kcal, and protein source, GMP or casein. Mice were randomized to one of the 4 diets, 60%-fat casein, 60%-fat GMP, 13%-fat casein, or 13%-fat GMP, after 4 days of acclimatization to the housing facility. Mice had free access to food and water and the facility was maintained at 22°C on a 12:12-h light-dark cycle. The high-fat diets energy from fat was sourced from combined soybean oil and lard, and the control diets energy from fat was sourced by soybean oil (Teklad, Madison, WI: TD140446-TD140449). The protein source in the casein diet was provided by 20% (wt/wt) casein plus 0.3% L-cystine and in the GMP diets 20% GMP (LACPORDEN CGMP-10, Arla Foods Ingredients, Viby J, Denmark) plus 1.5 times the NRC requirement for 6 limiting amino acids in order to provide a complete protein source [29]. The GMP diets were also supplemented with an additional 0.8% L-leucine and 0.15% L-methionine to match the amino acid profile of casein with regards to leucine and methionine. The analyzed amino acid profile of the diets is shown in Table 1. All 4 diets were supplemented with approximately 10% more than National Research Council requirements for calcium, phosphorous, and magnesium to optimize bone growth and the micronutrient content of the diets was constant. Mice were fed the experimental diets from weaning through adulthood (3 to 32 weeks of age), which resulted in the mice being fed for 29 weeks (n = 44 mice). The mice were weighed between 0900–1100 hr five times per week for the first 4 weeks of feeding and then once per week through the end of the study. Food intake was measured at the same time as body weight was measured, by subtracting the weight of food left in the cage from the original weight of food that was added to the cage.

**Table 1:** Complete amino acid analysis of the diets.

	60% Fat	60% Fat	13% Fat	13% Fat
Amino Acid	Casein	GMP	Casein	GMP
g/kg diet				
Alanine	5.5	9.2	5.6	9.2
Arginine	6.2	5.0	6.3	4.4
Aspartic acid	12.8	15.8	12.9	15.8
Cysteine	3.3	3.0	3.2	3.4
Glutamic acid	37.2	31.0	38.4	31.9
Glycine	3.5	2.4	3.5	2.3
Histidine*	5.5	2.3	5.5	2.5
Isoleucine*	9.4	15.1	9.2	14.9
Leucine*	17.0	15.6	16.9	15.9
Lysine*	14.6	12.1	14.5	11.9
Methionine*	5.1	4.8	5.0	4.4
Phenylalanine*	9.1	8.9	9.1	8.7
Proline	18.6	16.9	18.5	16.9
Serine	9.5	10.4	9.5	10.3
Threonine*	7.7	22.7	7.6	22.4
Tryptophan*	2.7	1.6	2.6	2.0
Tyrosine*	7.8	7.4	8.0	6.9
Valine*	11.7	12.9	11.6	12.7

The diets provided 60% Kcal from Fat (high-fat) or 13% Kcal from Fat (control) and either GMP or casein as the primary source of dietary protein.

* Indispensable amino acids

Dual-energy x-ray absorptiometry (DXA) with PIXImus software version 2.10 (GE/Lunar Corp, Madison, WI) was performed to obtain fat mass and fat-free mass of each mouse as well as in vivo whole body, femoral, and lumbar spinal BMD and BMC [17]. Following anesthesia with isoflurane via an anesthesia machine (IsoFlo, Abbott Laboratories, North Chicago, IL), mice were placed prone on the scanner bed with the limbs and tail stretched away from the body. One scan per mouse was performed at 4 different time points: 4, 8, 16, and 25 week after ingesting the experimental diets. The analysis of each scan excluded the head and provided a serial assessment of fat-free mass, fat mass, BMD, and BMC.

After being fed the experimental diets for 27 weeks, glucose tolerance tests were performed on mice fasted for 12 hours and insulin tolerances tests were performed on mice fasted for 4 hours. Animals were administrated with either 2 g/kg fat-free mass of glucose or 0.75 U/kg fat-free mass of human insulin (Novo Nordisk) by oral gavage or intraperitoneal injection, respectively. Blood samples were collected from the tail at *t* = 0, 15, 30, 60, 90, and 120 min to determine blood glucose concentrations.

Mice were anesthetized using isoflurane via an anesthesia machine and euthanized by exsanguination at 32 weeks of age after being fasted for 4–7 hr. Blood was collected by cardiac puncture into syringes containing a final concentration of 2.7 mmol/L EDTA and plasma was isolated by centrifugation at 4°C. Liver, heart, gastrocnemius muscle, intrascapular brown fat pad (brown adipose tissue, BAT), inguinal fat pad (used to determine subcutaneous fat), and gonadal fat pad (used to determine visceral fat) were dissected and weighed; ~100 mg was removed, homogenized, and used immediately for fatty acid oxidation analysis. The rest was snap frozen and stored and -80°C to be used for future analysis. Kidney, brain, and spleen were dissected and weighed. After euthanasia, both femora were dissected free of soft tissue, wrapped in phosphate buffered saline-saturated gauze, and stored at -80°C.

### Analytical Procedures

#### Ex Vivo Fatty Acid Oxidation

Liver, heart, gastrocnemius muscle, gonadal and inguinal white adipose tissues, and intrascapular BAT were collected, and homogenized in a Tris-sucrose-EDTA buffer and processed as described previously [30]. The homogenate as centrifuged at 420 x g for 10 min. at 4°C to separate the crude mitochondrial fraction. The supernatant containing mitochondria was incubated for 1 hr in the presence of 300 μM palmitic acid with 0.4 μCi 1-^14^C-palmitic acid. 200 μL of perchloric acid was added to the homogenate causing any long-chain fatty acids to precipitate out from the reaction mixture. Filter paper coated with hyamine hydroxide captures CO_2_ released after the addition of perchloric acid. The ^14^C-containing acid soluble metabolites and trapped CO_2_ were measured in a liquid scintillation counter. Total fatty acid oxidation reflects the sum of the acid-soluble metabolite and CO_2_ fractions. Tissue protein levels were measured using BCA from Pierce (Rockford, IL). Data are presented as total oxidative rate expressed as nmol/mg protein/hr and nmol/g tissue/hr.

#### Real Time Quantitative PCR Analysis

To assess levels of carnitine palmitoyltransferase I-α (CPT1-α), carnitine palmitoyltransferase I-β (CPT1-β), lipoprotein lipase (LPL), peroxisome proliferator-activated receptor gamma coactivator 1- α (PGC-1α), peroxisome proliferator-activated receptor alpha (PPAR-α), peroxisome proliferator-activated receptor gamma (PPAR-γ), sterol regulatory element-binding protein 1 (SREBP-1c), and uncoupling protein 1 (UCP1) mRNA in visceral and subcutaneous fat, the tissues were homogenized and RNA extracted using the Aurum Total RNA Mini kit (Bio-Rad) and purified by on-column digestion of DNA with DNase I to eliminate residual genomic DNA. Total RNA was used for cDNA synthesis (High-Capacity cDNA Reverse Transcriptase Kit, Applied Biosystems). Real time quantitative PCR was performed with LightCycler 480 SYBR Green I Master mix and analyzed with the Light Cycler 480 real-time PCR machine (Roche Applied Science, Indianapolis, IN). Cyclophilin expression was used as an internal control, and the primer sequences of the cyclophilin gene were F: TGGAGAGCACCAAGACAGACA and R:TGCCGGAGTCGACAATGAT. The primers used for qPCR were as follows: *CPT1-α-*F: AGACTTCCAACGCATGACAGCACTG, *CPT1-α-*R: CTCGGCCCCGCAGGTAGATG; *CPT1-β*-F: CGAGAGGGGCGGACTGAGACTG, *CPT1-β*-R: GGCTAGGCGGTACATGTTTTGGTG; *LPL*-F:TGCTCCCAACAATATAAGACTCC, *LPL*-R: AAGGCCAGGTGTTTCAATC; *PGC-1α*-F: AGCCGTGACACTGACAACGAG, *PGC-1α*-R: GCTGCATGGTTCTGAGTGCTAAG; *PPAR-α*-F: ATGGGGGTGATCGGAGGCTAATAG, *PPAR-α*-R: GGGTGGCAGGAAGGGAACAGAC; *PPAR-γ*-F: TCAGGTTTGGGCGGATGC, *PPAR-γ*-R: TCAGCGGGAAGGACTTTATGTATG; *SREBP-1c*-F: GGAGCCATGGATTGCACATT, *SREBP-*1c-R: GGCCCGGGAAGTCACTGT; *UCP1*-F: TCCTAGGGACCATCACCACC, *UCP1*-R: GCAGGCAGACCGCTGTACA. The 2^−ΔΔCt^ method was used to calculate the -fold change in gene expression [31].

#### Liver Triglycerides

Approximately 100 mg of liver tissue was homogenized in 1 mL of 5% Tergitol type NP-40/ddH2O solution using a Dounce homogenizer. Samples were slowly heated in an 80–100°C water bath to solubilize all triglyceride. Triglycerides were then quantified using a colorimetric assay (Abcam, San Francisco, CA).

#### Plasma Analyses

A colorimetric assay was used to measure plasma levels of non-esterified fatty acids (NEFA) and plasma glucose (Abcam). Plasma leptin, adiponectin, ghrelin, gastric inhibitory polypeptide, glucagon-like peptide-1, plasminogen activator inhibitor-1, resistin, glucagon, and insulin were measured using the mouse Bio-Plex diabetes multiplex bead-based assay according to manufacture protocol (Bio-Rad, Hercules, CA). Cytokines were measured using the Bio-Plex multiplex mouse cytokine 23-plex assay according to manufacture protocol (Bio-Rad).

### Bone Size and Biomechanical Testing

Femora specimens were subjected to two freeze thaw cycles, one prior to DXA, and the second prior to biomechanical testing. Areal BMD of isolated femora was measured by DXA as described previously [32]. Prior to biomechanical analysis, femora were gradually warmed by placing them at 4°C for at least 12 hrs, and then gradually allowed to come to room temperature prior to analysis. Femur length was measured before fracture by use of Vernier calipers to measure the distance between the greater trochanter and the medial condyle. Femoral diaphysis biomechanical performance was assessed by 3-point bending test as previously described [22]. Periosteal perimeter, cortical cross-sectional area, outer and inner major and minor axis lengths, shape factor, and cross-sectional moment of inertia was obtained from digital photographs of the fracture plane [33]. Right and left femora were averaged together for final analysis.

### Principal Component Analysis

The following input variables were included in principal component analysis (PCA): Body mass, femoral BMD, post-yield deflection, total deflection, yield load, maximum load, energy, stiffness, femoral cross-sectional area, femoral periosteal perimeter, femoral inner major and minor axis lengths, femoral outer major and minor axis lengths, femoral diaphyseal shape factor, and femoral diaphyseal cross-sectional moment of inertia. The SAS function Proc Princomp (SAS institute, Cary, NC) was used to perform the PCA [34–36]. Body weight did not impact the PCA; and thus, data were not corrected for body weight. Further analysis was performed for the principal components (PCs) with Eigenvalues ≥ 1. Multiplying each PCs Eigenvector by the animal’s parameter vector gave us the PC value for each animal.

### Liver Metabolomics

The non-targeted global metabolomic analysis was carried out by Metabolon, Inc. (Durham, NC). Liver samples from mice that were fed the 13%-fat casein and the 13%-fat GMP diets were prepared using the automated MicroLab STAR system (Hamilton Company). Samples were either run on ultra-performance liquid chromatography (UPLC)-MS/MS or GC-MS. The LC/MS portion of the platform was based on a Waters ACQUITY UPLC and a Thermo Scientific Q-Exactive high resolution/accurate mass spectrometer interfaced with a heated electrospray ionization source and Orbitrap mass analyzer operated at 35,000 mass resolution. Samples were analyzed on a Thermo-Finnigan Trace DSQ fast-scanning single-quadrupole mass spectrometer using electron impact ionization. Raw data was extracted, and compounds were identified by comparison to purified standards using Metabolon’s proprietary hardware and software.

### Statistical Analysis

Data were analyzed by two-way ANOVA using generalized linear model to identify the main treatment effects of protein source and fat content of the diet as well as their interaction. Differences between the treatment groups were detected by using a protected Fisher's least significant difference test (SAS Institute, 2007, Cary, NC). Data transformations were performed where appropriate to fit assumptions of normality and equal variance prior to statistical analysis. Data are presented as means ± SE. *P* values < 0.05 are considered significant.

Changes in body weight, food intake, and repeated DXA time points were analyzed using a repeated-measures model within PROC MIXED. The model included the fixed effects of protein source, fat content, and their interaction, as well as the factor time. To account for autocorrelated errors, an autoregressive error structure was included, and the Kenward-Roger method was used to compute the denominator degrees of freedom for the tests of the fixed effects.

Femoral cross section measurements and biomechanical data were adjusted for the animal’s body mass by including body mass as a covariate. When body mass was not a significant predictor for a parameter, the term was removed and results from a subsequent two-way ANOVA are presented. Biomechanical data are an average of the right and left femur. Pearson’s correlation analysis was used to evaluate the relationships among fat pad weights, gastrocnemius muscle weights, fat-free mass, and fat mass with bone measurements, including, BMC, BMD, yield load, max load, post-yield displacement, energy to fracture, cross-sectional area, and periosteal perimeter.

## Results

### Growth Rate, Food Intake, and Body Composition

**Fig 1:**
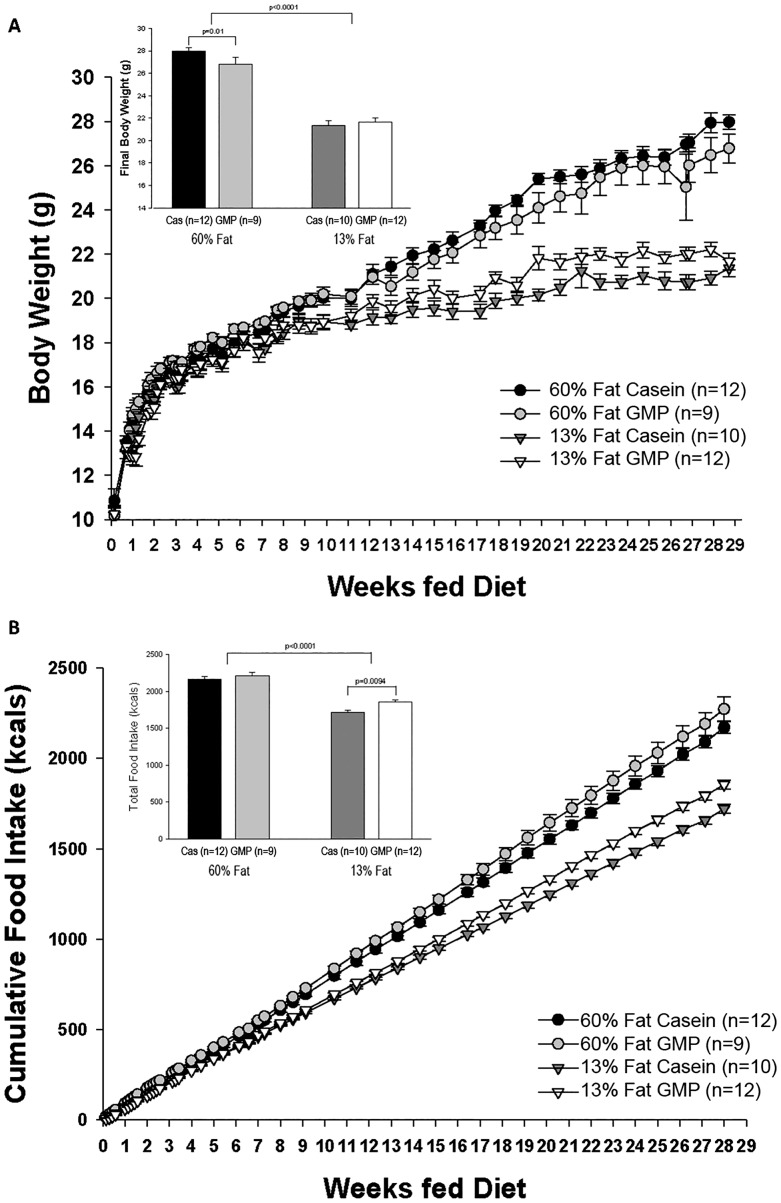
Body weight and food intake. Change in body weight (A) and cumulative food intake over time (B) of female mice fed 60%-fat casein, 60%-fat GMP, 13%-fat casein, and 13%-fat GMP diets from weaning (3week) through 32 weeks of age. Repeated measures analysis showed a significant protein x fat x time interaction for body weight (A). The inset histogram displays final body weight of the female mice. Mice fed the high-fat diets weighed significantly more at the end of the study (p<0.0001) compared to mice fed the control diets. Mice fed the high-fat GMP diet weighed significantly less at the end of the study compared to mice fed the high-fat casein diet (p = 0.01). Repeated measures analysis indicated a significant protein x fat x time interaction for cumulative food intake (B). The inset histogram shows total caloric intake throughout the study. Mice fed the high-fat diets consumed significantly more kcals throughout the study than mice fed the control diets (p<0.0001). Mice fed the 13%-fat GMP diet had a greater caloric intake compared to mice fed the 13%-fat casein diet.

Growth curves showing change in body weight for female mice from weaning (3 weeks of age) to 32 weeks of age are shown in Fig 1A. Beginning at 13 weeks of feeding diet, mice fed either of the high-fat diets showed a significantly greater accretion of body weight compared to mice fed the control diets and this was maintained through the end of the study. Final body weight was increased by 23% in mice fed the high-fat diets compared to mice fed the control diets (27.4 ± 0.52 vs 21.1 ± 0.26; p<0.0001). Mice fed the high-fat GMP diet had a significantly lower final body weight compared to mice fed the high-fat casein diet (p = 0.01).

Cumulative food intake of mice fed the four defined diets is shown in Fig 1B. Starting at week 9 of being fed the diets, mice fed the two high-fat diets consumed more energy (kcals) throughout the study compared to mice fed the control diets. Calculating total food intake from the entirety of the study, mice fed the high-fat diets consumed 18% greater total energy compared to mice fed the control diets (2189 ± 27 vs 1795 ± 23, p<0.0001). Mice fed the 13%-fat GMP diet consumed significantly more total energy compared to mice fed the 13%-fat casein diet, 1856 ± 25 vs 1721 ± 25, respectively (p = 0.0094); although body weight was not significantly different. Feed or energy efficiency was calculated as kcal of body weight gained (4 kcal x g fat-free mass + 9 kcal x g fat mass) divided by kcal of food consumed. Mice fed the high-fat diets had 10% greater feed efficiency than mice fed the control diets (p<0.009).

**Fig 2:**
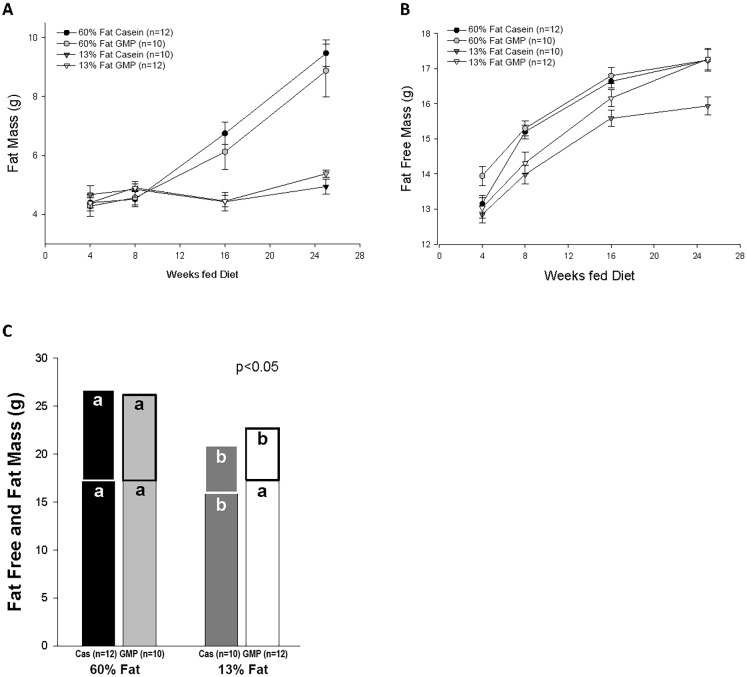
Body composition of female mice. Body composition of fat mass and fat-free mass over time determined by dual-energy X-ray absorptiometry in mice fed 60%-fat casein, 60%-fat GMP, 13%-fat casein, and 13%-fat GMP diets (A and B). Final composition of fat-free and fat-mass was assessed (C). ^a,b^Means with different superscripted letters are significantly different (p < 0.05). Repeated measures analysis of fat mass over time showed a significant fat*time interaction (p<0.0001). Repeated measures analysis of fat-free mass over time showed a significant prot*fat*time interaction (p<0.0001). There was a significant fat effect for final fat mass where mice fed the high-fat diets had greater fat mass than mice fed the control diets (C). There was a significant protein*fat interaction for final fat-free mass where mice fed the 13%-fat casein diet had a lesser fat-free mass than mice fed the other three diets. Nos. in parentheses indicate sample size.

Composition of fat-free and fat mass as determined by DXA after feeding the experimental diets for 4, 8, 16, and 25 weeks is shown in Fig 2. Mice fed the high-fat diets had a significant increase in fat mass compared to mice fed the control diets that was first shown at 16 weeks on diet, Fig 2A. Mice fed the 60%-fat casein, 60%-fat GMP, and 13%-fat GMP diets accrued more fat-free mass throughout the study than mice fed the 13%-fat casein diet, Fig 2B. At the final DXA time point after 25 weeks of feeding diet, there was a significant fat effect where mice fed the high-fat diets had a significantly greater fat mass than mice fed the control diets, 9.2 ± 0.5 vs 5.2 ± 0.1, respectively (p<0.0001), Fig 2C. There was a significant protein x fat interaction for fat-free mass at the final DXA time point (p = 0.034) where mice fed the high-fat casein and high-fat GMP diets had significantly greater fat-free mass than mice fed the control casein diet. This increase in fat-free mass, which includes both bone and lean mass, is needed to help carry the excess load of the greater fat mass induced by high-fat feeding [37]. Interestingly, we also saw significantly greater fat-free mass in mice fed the 13%-fat GMP diet compared to mice fed the 13%-fat casein diet.

### High-Fat Diets Did Not Lead to Glucose Intolerance in Female Mice

**Fig 3:**
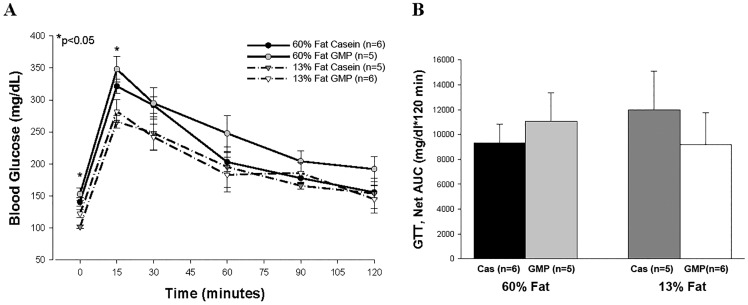
Glucose tolerance test. Glucose tolerance tests were performed on female mice fed 60%-fat casein, 60%-fat GMP, 13%-fat casein, or 13%-fat GMP diets after ingesting the diet for 27 weeks (A). Glucose was administered by oral gavage after an overnight fast (12h). B, net area under the curve (AUC) of the GTT displayed in A. The data are expressed as means ± SE. Nos. in parentheses indicate sample size. *, p<0.05.

Mice fed the high-fat diets for 27 weeks had significantly higher blood glucose concentrations at baseline and the fifteen-min time point during the oral glucose tolerance test (OGTT) compared to mice fed the control diets, as shown in Fig 3A. However, area under the curve was not significantly different between any of the four dietary treatments (Fig 3B). Similar results were seen in the insulin tolerance test as mice fed the high-fat diets had greater baseline blood glucose values, but no differences were seen over time between the four diets, S1 Fig. Plasma insulin concentration at the time of euthanasia was also not significantly different amongst the four diets (60%-fat casein, 1.94 ng/mL ± 0.15; 60%-fat GMP, 1.98 ng/mL ± 0.18; 13%-fat casein, 2.60 ng/mL ± 0.36; 13%-fat GMP, 1.98 ± 0.17), suggesting that the mice did not display glucose or insulin intolerance. This is not surprising as other investigators have shown that estrogen in female mice is protective against impaired glucose tolerance [38].

### High-Fat Diets Increase Adiposity and Ex Vivo Fatty Acid Oxidation in Muscle and Brown Fat

**Fig 4:**
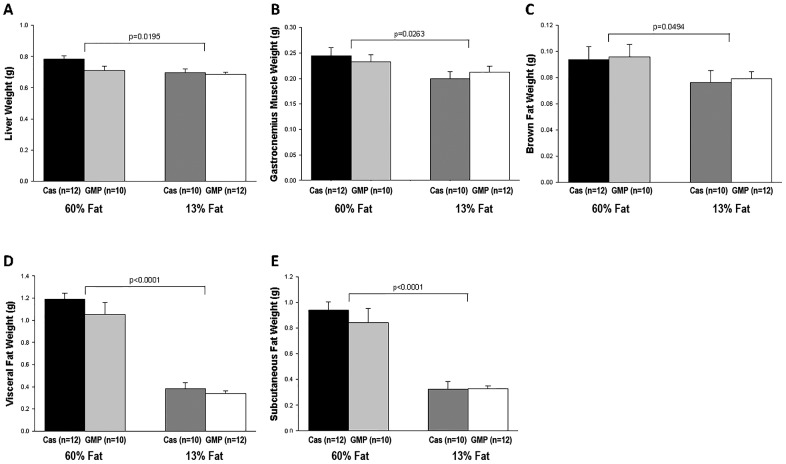
Fatty acid oxidation tissue weights. Liver weight (A), gastrocnemius muscle weight (B), brown fat weight (C), visceral fat weight (D), and subcutaneous fat weight (E) of female mice fed 60%-fat casein, 60%-fat GMP, 13%-fat casein, and 13%-fat GMP diets. Values are means ± SE; nos. in parentheses indicate sample size. There were significant fat effects for liver weight, gastrocnemius muscle weight, brown fat weight, visceral fat weight, and subcutaneous fat weight where mice fed the high-fat diets had heavier livers, gastrocnemius muscles, brown fat, visceral fat, and subcutaneous fat than mice that were fed the control diets.

Mass of liver, gastrocnemius muscle, and brown and white adipose tissue were significantly increased in mice fed the high-fat diets compared to mice fed the low-fat diets, as shown in Fig 4. For BAT, the fat pad removed was the intrascapular brown fat pad and this was significantly larger in mice fed the high-fat diets compared to mice fed the control diets, .095 ± .007 vs .078 ± .005 respectively (p = 0.0494). Two fat pads were removed to examine white adipose tissue weights in these mice, the gonadal fat pad used to examine visceral fat, and the inguinal fat pad used to examine subcutaneous fat. For visceral fat, mice fed the high-fat diets had a 3-fold greater fat pad weight compared to mice fed the control diets (p<0.0001). Consistent with greater visceral fat, mice fed the high-fat diets had a 2.7-fold greater subcutaneous fat pad weight than mice fed the control diets (p<0.0001).

**Fig 5:**
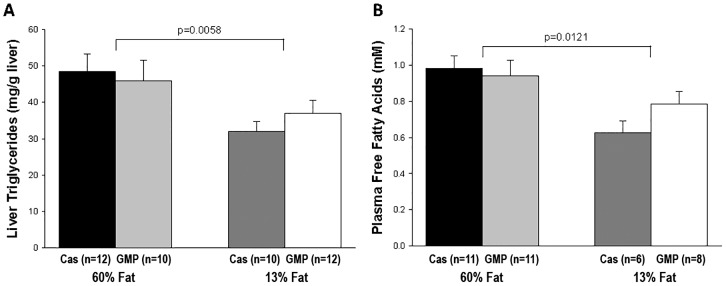
Concentrations of liver triglycerides and plasma free fatty acids. Concentrations of liver triglycerides (A) and plasma free fatty acids (B) in female mice fed 60%-fat casein, 60%-fat GMP, 13%-fat casein, and 13%-fat GMP diets. Values are means ± SE; nos. in parentheses indicate sample size. Concentration of liver triglycerides showed a significant fat effect where mice fed the high-fat diets had significantly greater liver triglycerides than mice fed the control diets (p = 0.0058). Mice fed the high-fat diets had significantly greater plasma free fatty acids than mice fed the control diets (p = 0.0121).

Liver triglyceride concentration was significantly increased in mice fed the high-fat diets compared to mice fed the control diets, 47.3 ± 3.6 mg/g liver vs 34.7 ± 2.4 mg/g liver (p = 0.0058), Fig 5A. Mice fed the high-fat diets also had a significant increase (p = 0.0121) in the concentration of plasma free fatty acids compared to mice fed the control diets as shown in Fig 5B.

**Fig 6:**
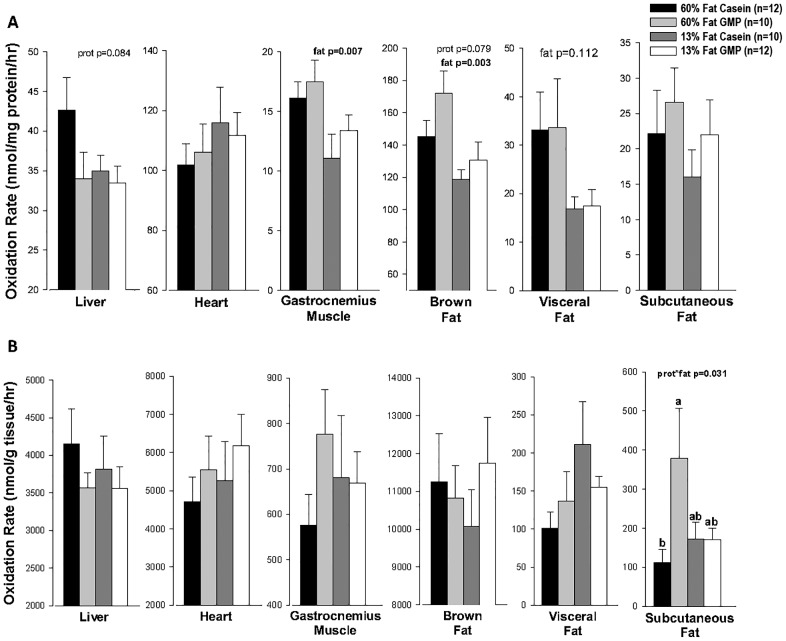
Total Fatty Acid Oxidation Rates. Total oxidation rate per mg protein (A) and per g tissue (B) in liver, heart, gastrocnemius muscle, brown fat, visceral fat, and subcutaneous fat tissues from female mice fed 60%-fat casein, 60%-fat GMP, 13%-fat casein, and 13%-fat GMP diets. Total fatty acid oxidation rate represents the sum of nanomoles of palmitate oxidized to CO_2_ and acid-soluble metabolites per hour. ^a,b^Means with different superscripted letters are significantly different (p < 0.05). Values are means ± SE; nos. in parentheses indicate sample size. There was a significant fat effect in oxidation rate per mg protein on gastrocnemius muscle and brown fat tissue where mice fed the high-fat diets had greater oxidation rates than mice fed the control diets (A). In subcutaneous fat tissue, there was a significant prot*fat interaction of total fatty acid oxidation rate per g tissue where mice fed the 60%-fat GMP diet had increased fat oxidized compared to mice fed the 60%-fat casein diet (B).

Ex vivo fatty acid oxidation was assessed in liver, heart, gastrocnemius muscle, brown fat, visceral fat, and subcutaneous fat to examine how fat and protein content of the diet alters oxidative capacity when substrate is not limiting. Total oxidation, the sum of complete oxidation to CO_2_ and incomplete oxidation in the acid-soluble metabolites, of each tissue is summarized in Fig 6. A significant effect of fat was seen in the gastrocnemius muscle where the high-fat diets increased the oxidation rate compared to the control diets by approximately 36%, 16.75 ± 1.10 vs 12.35 ± 1.15 nmol per mg protein per hour respectively. Mice fed the high-fat diets also had a significant increase (p = 0.003) in oxidation rate in the brown fat pad compared to mice fed the control diets. Given the larger total muscle mass relative to fat mass, high-fat feeding primarily increased oxidative capacity in muscle. No increase in fatty acid oxidative capacity due to high-fat feeding was seen in liver, heart, or subcutaneous adipose tissue. There was a trend for greater fatty acid oxidation with high-fat feeding in visceral adipose tissue (p = 0.112).

### GMP Increases Ex Vivo Fatty Acid Oxidation in Subcutaneous Fat and Brown Fat

There was a significant protein by fat interaction in subcutaneous fat tissue primarily driven by mice fed the high-fat GMP diet, which showed a 3-fold greater rate of total fat oxidation compared with mice fed the high-fat casein diet (379 ± 128 vs 112 ± 34 nmol per gram tissue), Fig 6. There was a trend for GMP to increase the rate of total fat oxidation in brown fat compared with casein (149.4 ± 9.7 vs 133.1 ± 6.6 nmol per mg protein per hour, p = 0.079).

### GMP and High-Fat Diets Alter Appetite Hormones & Expression of Fatty Acid Oxidation Genes

**Fig 7:**
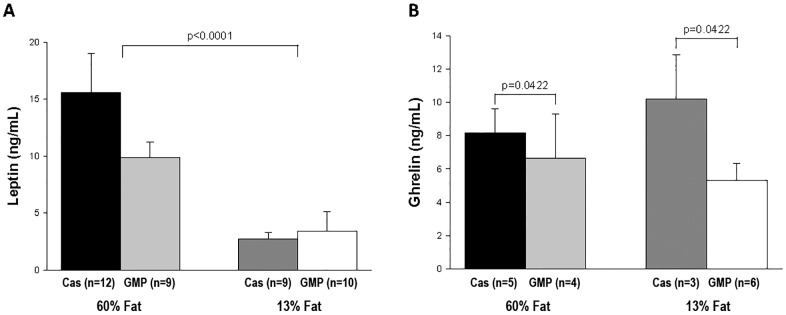
Plasma leptin and ghrelin concentrations. Plasma concentrations of leptin (A) and ghrelin (B) in female mice fed 60%-fat casein, 60%-fat GMP, 13%-fat casein, and 13%-fat GMP diets. Values are means ± SE; nos. in parentheses indicate sample size. There was a significant fat effect in plasma leptin where mice fed the high-fat diets had a significantly greater leptin concentration than mice fed the control diets (p<0.0001). There was a significant protein effect in plasma ghrelin where mice fed the GMP diets had a significantly greater ghrelin concentration than mice fed the casein diets (p = 0.0422).

Appetite hormones were measured in plasma to examine if these were impacting differences in food intake of the mice fed the four different diets. Plasma leptin concentration was significantly increased (p<0.0001) in mice fed the high-fat diets compared to mice fed the control diets, as shown in Fig 7A. Interestingly, mice fed the GMP diets had a significant decrease (p = 0.0422) in plasma ghrelin compared to mice fed the casein diets, 5.83 ± 0.68 vs 8.92 ± 1.28 ng/mL respectively, as shown in Fig 7B. Adiponectin was not significantly different among mice fed the four different diets; 60%-fat casein 5.12 ± 0.94 μg/mL, 60%-fat GMP 5.36 ± 0.53 μg/mL, 13%-fat casein 6.11 ± 0.55 μg/mL, and 13%-fat GMP 5.70 ± 0.87 μg/mL. Plasma gastric inhibitory polypeptide, glucagon-like peptide-1, plasminogen activator inhibitor-1, resistin, and glucagon showed no difference amongst the four dietary treatments, data shown in S1 Table.

**Fig 8:**
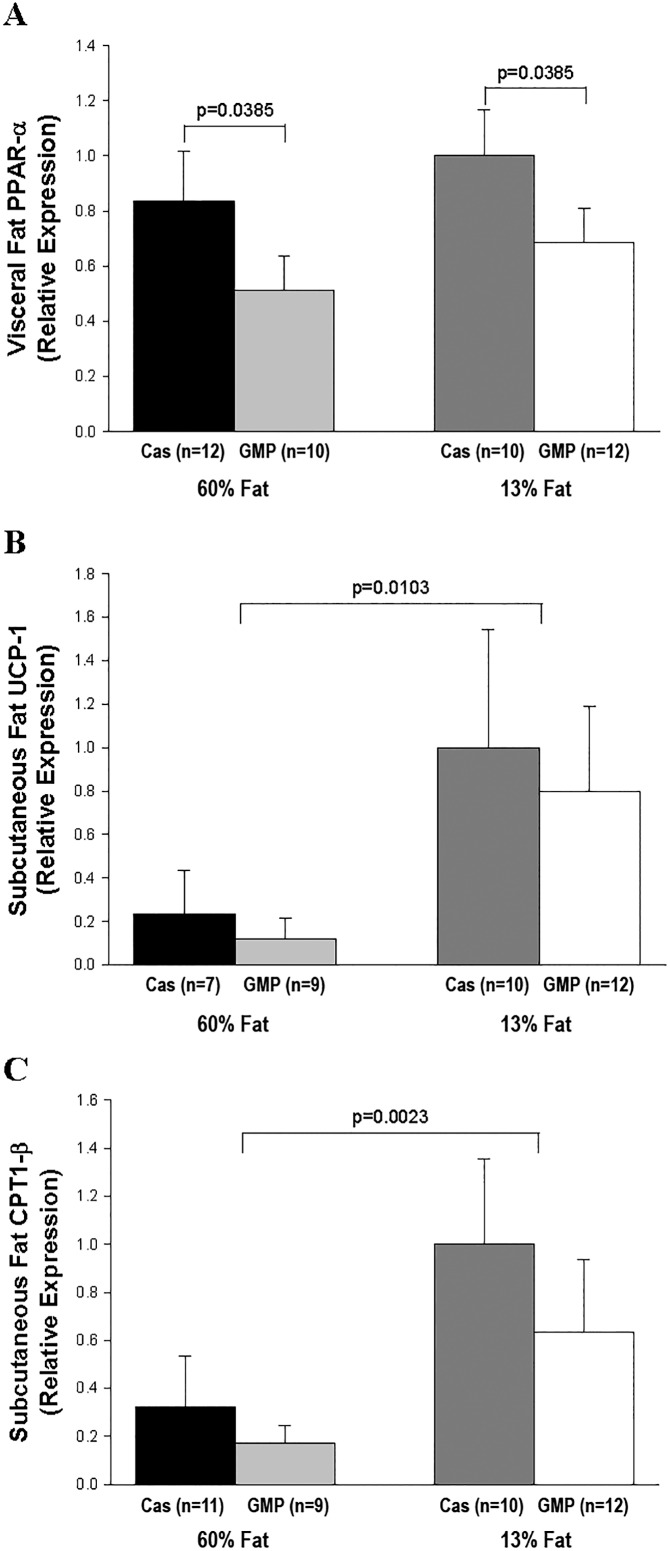
Relative expression of the genes PPAR-α, UCP-1, and CPT1-β. Relative expression of the genes PPAR-α (A) in visceral fat tissue and UCP-1 (B) and CPT1-β (C) in subcutaneous fat tissue by quantitative real-time PCR. The expression levels have been normalized with the average expression of the control gene cyclophilin. There was a significant protein effect in relative expression of PPAR-α where mice fed the GMP diets have reduced relative expression compared to mice fed the casein diets. There was a significant fat effect in relative expression of UCP-1 and CPT1-β where mice fed the high-fat diets have reduced relative expression compared to mice fed the control diets.

GMP significantly reduced (p = 0.0385) relative expression of PPAR-α in visceral fat tissue compared to mice that were fed the casein diets, Fig 8A. When examining expression of CPT1-α, CPT1-β, LPL, PGC1-α, PPAR-γ, SREBP1c, and UCP-1 in visceral fat tissue, there were no significant differences amongst mice fed the four different diets. In subcutaneous fat tissue, there were significant fat effects in the relative expression of CPT1-β and UCP-1, where mice fed the control diets had a greater relative expression than mice fed the high-fat diets, (p = 0.0023 and p = 0.0103, respectively), Fig 8B and 8C. Relative expression of PGC1-α in subcutaneous fat tissue was not different amongst mice fed the four different diets, 60%-fat casein 0.95 ± 0.21, 60%-fat GMP 0.89 ± 0.17, 13%-fat casein 1.00 ± 0.26, and 13%-fat GMP 0.62 ± 0.13 μg/mL.

### The GMP Diet Alters Threonine Metabolites in the Liver

**Fig 9:**
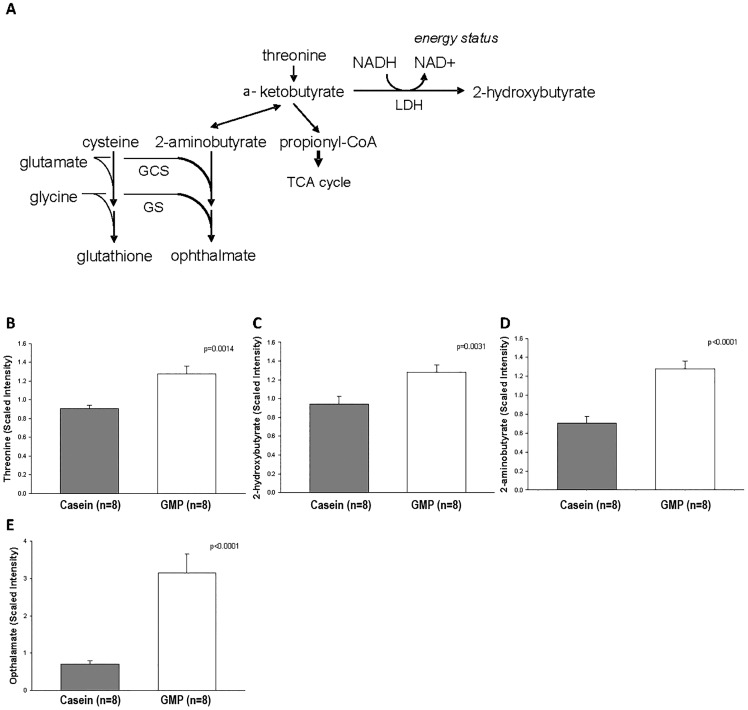
Liver threonine metabolites. A, metabolic pathway of threonine breakdown to its metabolites 2-aminobutyrate, propionyl-CoA, and 2-hydroxybutyrate. Mass spectrometry-scaled intensity of threonine (B), 2-hydroxybutyrate (C), 2-aminobutyrate (D), and opthalamate (E) metabolites in the livers of female mice fed the 13%-fat casein and 13%-fat GMP diets. The GMP diet significantly increased the presence of threonine, 2-hydroxybutyrate, 2-aminobutyrate, and opthalamate relative to the mice fed the casein diet.

Livers of mice fed the 13%-fat casein and GMP diets underwent metabolomics profiling of threonine metabolites as the concentration of threonine in GMP is 2-3-fold higher than in a typical protein, Table 1. Relative threonine concentration in liver tissue is significantly greater in mice fed the GMP diet compared to the casein diet (p = 0.0014) as shown in Fig 9. Threonine can be metabolized to 2-hydroxybutyrate, as shown in Fig 9, and consistent with a greater relative concentration of threonine, mice fed the GMP diet had a significant increase in relative liver concentration of 2-hydroxybutyrate compared to mice fed the casein diet (Fig 9C). Another metabolite of threonine, 2-aminobutyrate, is significantly higher in mice fed the GMP diet compared to mice fed the casein diet (p<0.0001), Fig 9D. Ophthalamate, an antioxidant synthesized from 2-aminobutyrate, was also significantly higher with GMP feeding, Fig 9E.

### In Vivo Bone Mineral Content and Density

After being fed the diets for 25 weeks, DXA scans were used to analyze total animal, femoral, and lumbar spine bone mineral content (BMC) and density, as shown in Table 2. For total animal BMC, there was a significant protein effect where mice fed the GMP diets had greater BMC than mice fed the casein diets. Mice fed the high-fat diets had a significantly greater total animal bone mineral density (BMD) than mice fed the control diets. When solely examining the femora, there was a significant fat effect where mice fed the high-fat diets had greater femoral BMC and BMD than mice fed the control diets. There were no significant differences in BMC and BMD of the lumbar spine among the four treatment groups.

**Table 2:** In vivo DXA measurements.

	60%-Fat	60%-Fat	13%-Fat	13%-Fat
Variable	Casein	GMP	Casein	GMP
*N*	12	10	10	12
Total Animal BMC (mg)^a^	384 ± 9	401 ± 10	364 ± 6	396 ± 6
Total Animal BMD (mg/cm^2^)^b^	54.2 ± 0.5	53.7 ± 0.7	51.2 ± 0.5	52.4 ± 0.4
Femur BMC (mg)^b^	25.0 ± 0.5	25.2 ± 0.7	23.4 ± 0.2	24.9 ± 0.4
Femur BMD (mg/cm^2^)^b^	72.7 ± 0.9	70.4 ± 1.7	67.8 ± 0.6	69.7 ± 0.9
Lumbar Spine BMC (mg)	42.6 ± 1.3	43.0 ± 1.5	42.2 ± 1.0	44.8 ± 1.0
Lumbar Spine BMD (mg/cm^2^)	59.2 ± 1.2	59.6 ± 1.6	62.2 ± 1.0	60.5 ± 1.0

Values are means ± SE; *N*, no of mice; BMC, bone mineral content; BMD, bone mineral density.

^a^ protein effect,

^b^ fat effect,

**Total Animal BMC:** Mice fed the GMP diets had a greater BMC than mice fed the casein diets. **Total Animal BMD:** Mice fed the high-fat diets had a greater BMD than mice fed the control diets. **Femur BMC:** Mice fed the high-fat diets had a higher BMC than mice fed the control diets. **Femur BMD:** Mice fed the high-fat diets had a significantly greater BMD than mice fed the control diets.

### High-Fat Diets Increase Femoral Size, Strength, and Brittleness

The 3-point bending test measures 3 elements of biomechanical performance, strength, displacement, and absorbed energy. Strength is the load or force applied to the bone, displacement the amount the bone bends, and absorbed energy is total energy to fracture calculated from the area under the load-displacement curve, data shown in Table 3. Post fracture specimen geometry of the fracture plane is assessed with data shown in Table 4. The high-fat diets significantly increased the femoral cross-sectional area, but not the length of femora, compared to mice fed the control diets, Table 4.

**Table 3:** Force-displacement Curve Analysis showing whole bone biomechanical performance of femora.

	60%-Fat	60%-Fat	13%-Fat	13%-Fat
Genus	Casein	GMP	Casein	GMP
*N*	11	9	10	12
^t^PY disp (mm)^b^	0.32 ± 0.03	0.29 ± 0.03	0.41 ± 0.06	0.47 ± 0.05
^t^Total disp (mm)^b^	0.47 ± 0.03	0.46 ± 0.04	0.57 ± 0.06	0.61 ± 0.05
Stiffness (N/mm)^b^	131 ± 5	133 ± 5	118 ± 5	120 ± 5
Yield load (N)	14.7 ± 0.3	14.3 ± 0.5	13.8 ± 0.5	13.6 ± 0.3
Max load (N)^b^	18.8 ± 0.3	19.2 ± 0.4	17.4 ± 0.5	17.1 ± 0.5
Energy (N-mm)	6.16 ± 0.30	5.84 ± 0.42	6.40 ± 0.49	7.23 ± 0.54

Values are means ± SE of raw data; *N*, no of mice; PY, post-yield; disp, displacement; N, newtons

^t^Data transformed to satisfy assumptions of normality and variance.

^b^ fat effect,

**PY disp:** Control diet fed mice had greater PY disp than high-fat fed mice. The effect was driven by the higher PY disp seen with the 13%-fat GMP diet as the 13%-fat casein diet was not different from the high-fat diets. **Total disp:** Control diet fed mice had greater total disp than high-fat fed mice. **Stiffness:** High-fat fed mice had stiffer bones than mice fed the control diets. **Yield Load:** Mice fed the high-fat diets had a greater yield load than mice fed the control diets (p = 0.056). **Max load:** Mice fed the high-fat diets tolerated a greater maximum load before fracture compared to mice fed the control diets. **Energy:** It took more energy to fracture the femurs of mice fed the control diets compared to mice fed the high-fat diets (p = 0.08).

**Table 4:** Size, shape, and mineral content of mice femora.

	60%-Fat	60%-Fat	13%-Fat	13%-Fat
Variable	Casein	GMP	Casein	GMP
*N*	11	9	10	12
Length (mm)^a^	15.2 ± 0.1	15.3 ± 0.1	15.0 ± 0.1	15.3 ± 0.1
CSA (mm^2^)^b^	0.89 ± 0.02	0.88 ± 0.02	0.80 ± 0.02	0.86 ± 0.02
Perimeter (mm)^c^	5.05 ± 0.04	5.00 ± 0.05	4.91 ± 0.06	5.06 ± 0.04
Inner Minor Axis (mm)	0.80 ± 0.01	0.80 ± 0.01	0.81 ± 0.01	0.82 ± 0.01
Inner Major Axis (mm)	1.27 ± 0.02	1.26 ± 0.01	1.29 ± 0.02	1.30 ± 0.02
Outer Minor Axis (mm)	1.26 ± 0.01	1.25 ± 0.01	1.24 ± 0.01	1.26 ± 0.01
Outer Major Axis (mm)	1.78 ± 0.02	1.77 ± 0.02	1.73 ± 0.03	1.77 ± 0.01
Shape Factor (unitless)	1.42 ± 0.01	1.41 ± 0.02	1.39 ± 0.01	1.40 ± 0.01
CSMI (mm^4^)	0.13 ± 0.01	0.13 ± 0.01	0.12 ± 0.01	0.13 ± 0.01
BMD (mg/cm^2^)^b^	58.0 ± 0.8	59.2 ± 0.7	55.4 ± 0.6	57.0 ± 0.7
BMC (mg)^b^	30.3 ± 1.5	29.9 ± 1.1	26.1 ± 0.4	28.4 ± 1.1

Values are means ± SE of raw data; *N*, no of mice; CSA, cross sectional area; CSMI, cross sectional moment of inertia; BMD, areal bone mineral density; BMC, bone mineral content. ^t^Data transformed to satisfy assumptions of normality and variance.

^a^ protein effect,

^b^ fat effect,

^c^ protein by fat interaction effect.

**Length:** Mice fed the GMP diets had longer femora than mice fed the casein diets. **CSA:** Mice fed the high-fat diets had a greater CSA than mice fed the control diets. **Perimeter:** Mice fed the 13%-fat GMP diet and the high-fat casein diet had a larger perimeter than mice fed the 13%-fat casein diet. **BMD:** Mice fed the high-fat diets had higher BMD than mice fed the control diets. **BMC:** Mice fed the high-fat diets had greater BMC than mice fed the control diets.

**Fig 10:**
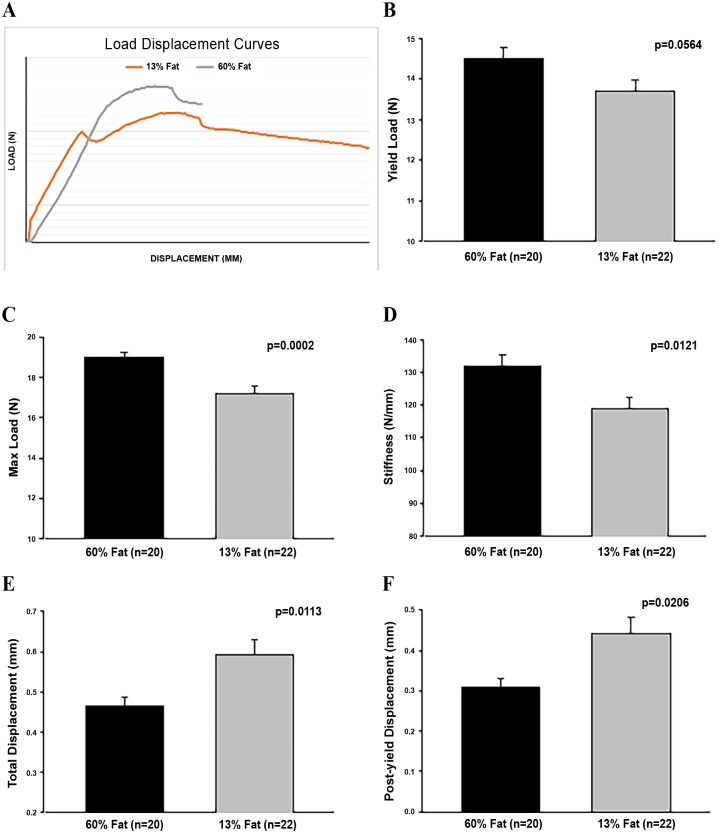
Bone measures in high-fat versus control diets. Representative load-displacement curves of female mice fed the high-fat and control diets (A). Effects in female mice fed high-fat and control diets for yield load (B), max load (C), stiffness (D), total displacement (E), post-yield displacement (F). Values are means ± SE; p-values represent main effect of fat. Sample size is shown in parenthesis. Mice fed the high-fat diets had increased femoral biomechanical performance values for yield load, max load, and stiffness compared to mice fed the control diets. Mice fed the high-fat diets had reduced femoral biomechanical performance values for total displacement and post-yield displacement compared to mice fed the control diets.

Representative load-displacement curves of mice fed the high-fat diet and mice fed the control diet are shown in Fig 10. Mice fed the high-fat diets had a higher yield load, which is the force required to begin permanently damaging the femur, and a significantly greater maximum load achieved before fracture compared to mice that were fed the control diets. Stiffness of the bone is also significantly greater in mice fed the high-fat diets compared to mice fed the control diets. In contrast, total displacement, the total amount of deformation by the femur before fracture, is significantly lower in mice fed the high-fat diets compared to mice fed the control diets. Post-yield displacement, a measure of ductility, is also significantly reduced in mice fed the high-fat diets, which signifies that mice fed the high-fat diets have more brittle bones. This data taken together suggests that mice fed a high-fat diet have stronger yet more brittle bones. Ex vivo DXA data supports greater mineralization of the femora as mice fed the high-fat diets had significantly greater BMD and BMC compared to mice fed the control diets, Table 4.

### Principal Components Analysis

**Fig 11:**
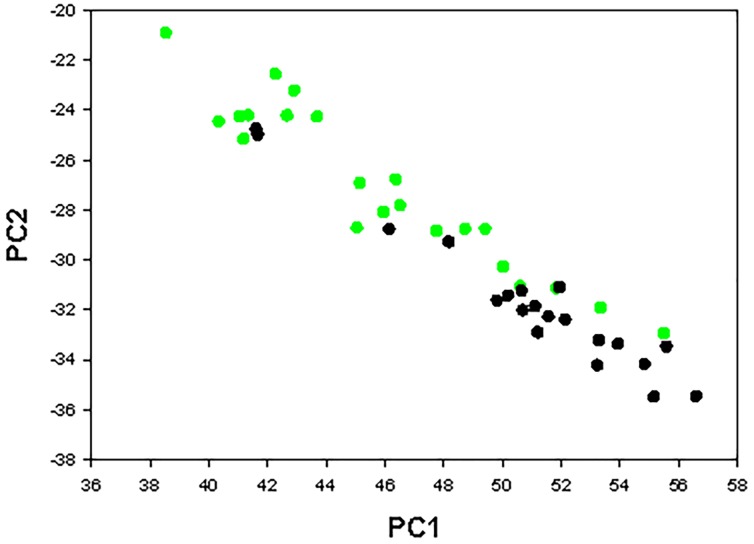
Principal Component Analysis Plot. Principal component plot of mice fed high-fat and control diets. Each dot represents an individual mouse. Mice fed the control diets are the green circles and mice fed the high-fat diets are the black circles. PC1 “Size-like” vs. PC2 “Ductility-like”.

The bone properties that were examined in this study are not completely independent as the correlation matrix among phenotypes shows in Table 5. To determine which properties contributed most to the bone phenotype, a principal components analysis (PCA) was performed on the raw data. PCA linearly transforms the raw data into an equal number of mutually orthogonal PC’s, which is a linear function of the raw data [39, 40]. Further analysis is continued on only those PC’s with an Eigenvalue greater than one. The analysis included 16 whole bone femoral measurements and this yielded four PC’s with an Eigenvalue >1, which, all together, accounted for 79% of the variance as shown in Table 6. The PC’s are comprised of all measures, but interpretations can be made based on the coefficients of the Eigenvectors, Table 7. PC1 is “size-like” as the major contributors are cross-sectional area, perimeter, outer minor axis, outer major axis, and CSMI. PC2 is “ductility-like” as post-yield deflection, total deflection, and energy to failure are the most prominent contributors. PC3 appears to be “shape-like” as the major contributor is shape factor. PC4 is the most difficult to interpret as body mass, max load, and energy are the major contributors. Consistent with the interpretations of the PC’s from the Eigenvectors, there are significant fat effects for all four PC’s, indicating that dietary fat content is the primary factor contributing to the phenotype seen in biomechanical performance of the femora, Table 8. The impact of high-fat feeding to increase femoral cross sectional area and decrease bone ductility, or increase bone brittleness, was especially dramatic, Fig 11.

**Table 5:** Correlations among phenotypes.

	**Mass**	**CSA**	**Perim**	**InMin**	**InMaj**	**OutMin**	**OutMaj**	**SF**	**CSMI**	**PYD**	**TD**	**Stiff**	**YLoad**	**MLoad**	**Energy**	**fBMD**
**Mass**	-	0.40	0.20	-0.19	-0.16	0.11	0.23	0.17	0.22	-0.30	-0.27	0.36	0.32	0.54	-0.14	0.43
**CSA**		-	0.86	-0.10	0.18	0.60	0.81	0.43	0.86	0.02	-0.02	0.47	0.33	0.61	0.34	0.60
**Perim**			-	0.23	0.49	0.74	0.91	0.42	0.89	0.12	0.06	0.38	0.31	0.43	0.36	0.43
**InMin**				-	0.29	0.52	0.09	-0.34	0.22	0.20	0.16	-0.11	-0.07	-0.17	0.14	-0.14
**InMaj**					-	0.36	0.39	0.17	0.49	0.24	0.22	0.03	0.10	-0.02	0.26	0.08
**OutMin**						-	0.55	-0.21	0.83	0.19	0.11	0.19	0.22	0.28	0.40	0.24
**OutMaj**							-	0.70	0.70	0.01	-0.04	0.44	0.31	0.45	0.24	0.42
**SF**								-	0.11	-0.15	-0.13	0.35	0.17	0.28	-0.07	0.30
**CSMI**									-	0.22	0.16	0.34	0.25	0.42	0.49	0.48
**PYD**										-	0.96	-0.26	-0.34	-0.40	0.90	-0.35
**TD**											-	-0.42	-0.30	-0.45	0.86	-0.41
**Stiff**												-	0.13	0.61	-0.11	0.63
**YLoad**													-	0.51	-0.15	0.23
**MLoad**														-	-0.12	0.70
**Energy**															-	-0.16
**fBMD**																-

Phenotypes are Mass, body mass; CSA, cortical cross-sectional area; Perim, periosteal perimeter; InMin, inner minor axis; InMaj, inner major axis; OutMin, outer minor axis; OutMaj, outer major axis; SF, shape factor; CSMI, cross-sectional moment of inertia; PYD, post-yield deflection; TD, total deflection; Stiff, stiffness; YLoad, yield load; MLoad, maximum load; Energy, energy to failure; fBMD, BMD by ex vivo DXA. Each cell shows R.

**Table 6:** Principal Component Analysis

	PC1	PC2	PC3	PC4
**Eigenvalue**	5.78	3.97	1.61	1.25
**Difference**	1.81	2.36	0.37	0.29
**r^2^**	0.361	0.248	0.101	0.078
**Cumulative r^2^**	0.361	0.610	0.710	0.788

Difference is subtraction of subsequent PC from former PC.

**Table 7:** Eigenvectors of Principal Components 1–4.

	PC1	PC2	PC3	PC4
Body mass	0.183	-0.203	0.052	0.411
Cross-sectional area	0.384	0.043	0.132	0.136
Perimeter	0.378	0.149	-0.027	-0.168
Inner minor axis	0.023	0.220	-0.554	-0.166
Inner major axis	0.151	0.219	-0.103	-0.483
Outer minor axis	0.275	0.222	-0.411	0.117
Outer major axis	0.363	0.063	0.142	-0.292
Shape factor	0.190	-0.113	0.517	-0.446
CSMI	0.357	0.198	-0.116	0.096
Post-yield deflection	-0.039	0.453	0.228	0.176
Total deflection	-0.068	0.444	0.250	0.156
Stiffness	0.253	-0.183	0.078	0.086
Yield load	0.185	-0.147	-0.152	-0.031
Max load	0.297	-0.226	-0.015	0.240
Energy	0.086	0.431	0.217	0.265
Femoral BMD	0.286	-0.195	0.010	0.155

CSMI, cross-sectional moment of inertia; BMD, bone mineral density.

**Table 8:** Principal component phenotypes of mice fed 60%-fat casein, 60%-fat GMP, 13%-fat casein, and13%-fat GMP diets.

	60%-Fat	60%-Fat	13%-Fat	13%-Fat
Variable	Casein	GMP	Casein	GMP
***N***	11	9	10	12
^t^**PC1**^b^	50.8 ± 1.3	51.2 ± 1.3	45.5 ± 1.5	46.3 ± 1.3
^t^**PC2**^b^	-31.5 ± 0.9	-31.9 ± 1.0	-27.0 ± 1.0	-27.1 ± 1.0
^t^**PC3**^b^	10.5 ± 0.4	10.6 ± 0.4	9.3 ± 0.5	9.8 ± 0.4
^t^**PC4**^b^	26.0 ± 0.5	25.8 ± 0.5	21.8 ± 0.7	22.4 ± 0.5

Values are means ± SE; *N*, no of mice; PC, principal component.

^t^ Data transformed to satisfy assumptions of normality and variance.

^b^ fat effect,

**PC1:** Mice fed the high-fat diets had a significant increase in their calculated PC1 “size-like” values compared to mice fed the control diets. **PC2:** Mice fed the high-fat diets had reduced “ductility-like” values compared to mice fed the control diets. P**C3:** Mice fed the high-fat diets had greater “shape-like” values than mice fed the control diets. **PC4:** Mice fed the high-fat diets had greater values than mice fed the control diets.

### GMP Increases Femoral Size, Total Bone Mineral Content, and Reduces Femoral Brittleness

**Fig 12:**
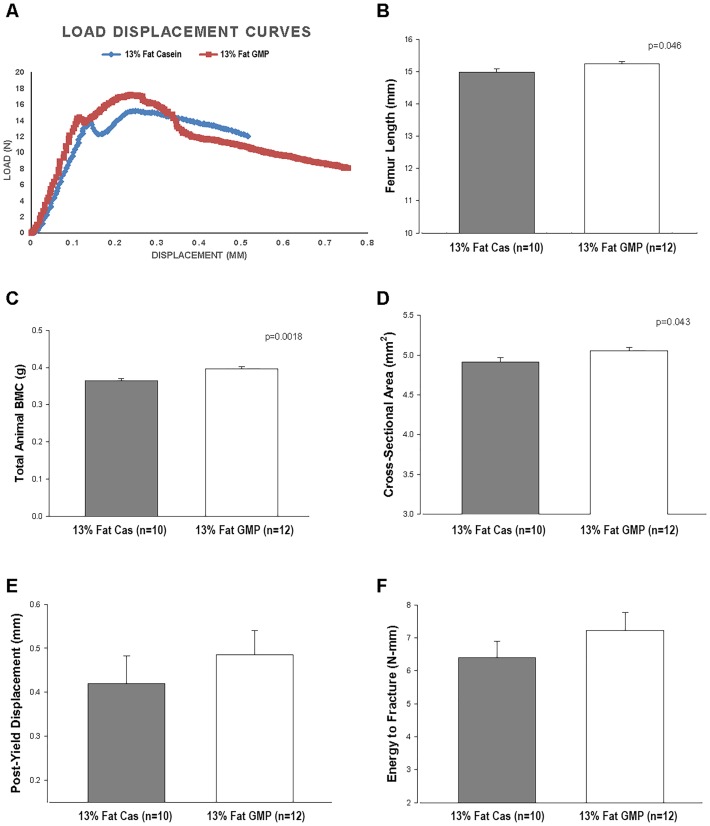
Bone measures of mice fed 13%-fat casein and 13%-fat GMP diets. Representative load-displacement curves of female mice fed the 13%-fat casein and 13%-fat GMP diets (A). Effects in female mice fed 13%-fat casein and 13%-fat GMP diets for femur length (B), total animal BMC (C), cross-sectional area (D), post-yield displacement (E), and energy to fracture (F). Values are means ± SE; p-values represent main effect of fat. Sample size is shown in parenthesis. Mice fed the 13%-fat GMP diet had significantly increased total animal BMC and femoral cross-sectional area and length compared to mice fed the 13%-fat casein diet. Mice fed the 13%-fat GMP diet had increased femoral biomechanical performance values for post-yield displacement and energy to fracture compared to mice fed the 13%-fat casein diet.

Mice fed the GMP diets had significantly longer femora (p = 0.0485) than mice fed the casein diets, Fig 12B. There was a significant protein by fat interaction when the femoral perimeter was measured, where mice fed the high-fat casein diet and 13%-fat GMP diet had significantly larger perimeters than mice fed the 13%-fat casein diet, Table 4. Mice fed the 13%-fat GMP diet had a significantly greater cross-sectional area than mice fed the 13%-fat casein diet, and this was not significantly different compared with mice fed the high-fat diets.

Mice fed the 13%-fat GMP diet had greater total animal BMC when compared to mice fed the 13%-fat casein diet, Fig 12C. The BMC in mice fed the 13%-fat GMP diets is similar to that of mice fed the high-fat diets. Mice fed the control diets had significantly greater post-yield displacement than mice fed the high-fat diets and this was driven by the 13%-fat GMP diet as mice that were fed the 13%-fat casein diet had a post-yield displacement that was not significantly different from mice fed the high-fat diets, Table 3. These data suggest that GMP is improving ductility of the bone, meaning that it is reducing the brittleness of the bone. Mice fed the 13%-fat GMP diet also had the greatest energy to fracture, calculated as the area under the load-displacement curve, compared to mice fed the other three diets, Fig 12F. This is a function of GMP reducing the brittleness of the bone.

### Both Fat-Free and Fat Mass Are Correlated with Bone Status

**Fig 13:**
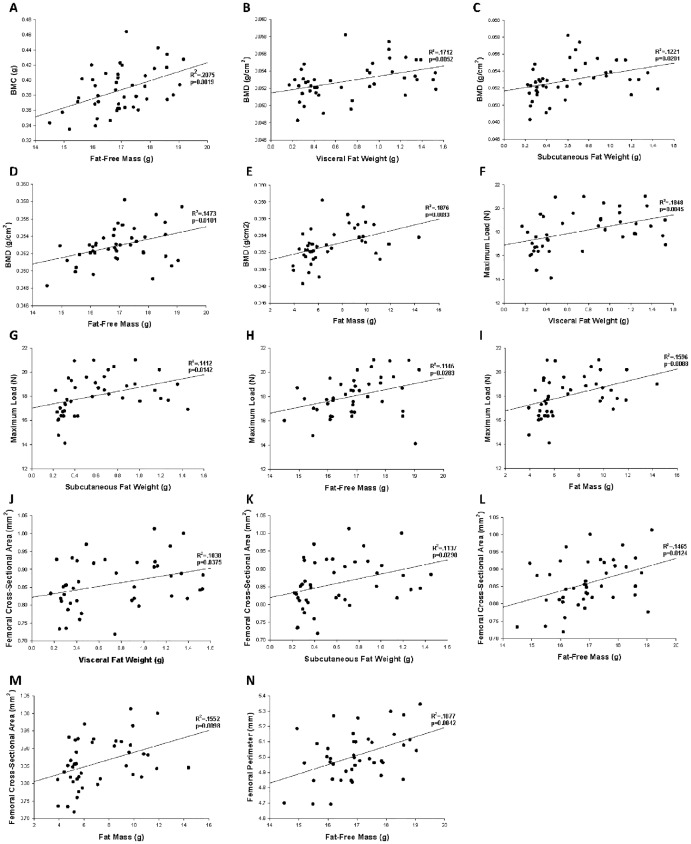
Reciprocal relations of body composition and bone measures. Reciprocal relations of fat-free mass, fat mass, visceral fat weight, and subcutaneous fat weight with measures of mineralization (A-E), strength (F-I), and cross-sectional dimensions (J-N) of the femur.

Correlational analysis was used to examine the relationship between markers of body composition, including visceral fat weight, subcutaneous fat weight, brown fat weight, gastrocnemius muscle weight, fat-free mass, and fat mass, and markers of bone status. Results are displayed in Table 9, and the significant relationships are shown in Fig 13. Fat-free mass had the greatest impact on mineralization of the bone as reflected in increased BMC and BMD with high-fat feeding, Fig 13A and 13D. Max load, reflective of the strength of the bone, is significantly related to visceral fat weight, subcutaneous fat weight, fat-free mass, and fat mass with the largest impacts coming from the weight of visceral and subcutaneous fat tissues. Bone size measures, cross-sectional area and perimeter, are significantly related to visceral fat weight, subcutaneous fat weight, fat-free mass, and fat mass. All markers of fat were negatively correlated with post-yield displacement, a measure of ductility. Post-yield displacement decreased as total fat mass, and the mass of subcutaneous, visceral and BAT increased (Table 9).

**Table 9:** Correlations between body composition and bone parameters.

Independent Variable		BMC (g)	BMD (g/mm^2^)	Yield Load (N)	Max Load (N)	Post-Yield Displacement (mm)	Energy (N-mm)	Cross-Sectional Area (mm^2^)	Perimeter (mm)
**Visceral Fat Weight (g)**	R^2^	0.004	0.171	0.086	0.185	0.070	0.023	0.104	0.013
	Regression coefficient ± SE	0.004 ± 0.010	0.002 ± 0.001*	0.884 ± 0.456	1.59 ± 0.53*	-0.095 ± 0.055	-0.530 ± 0.543	0.051 ± 0.024^§^	0.041 ± 0.057
**Subcutaneous Fat Weight (g)**	R^2^	0.001	0.122	0.068	0.141	0.085	0.027	0.114	0.016
	Regression coefficient ± SE	0.002 ± 0.013	0.002 ± 0.001*	0.980 ± 0.571	1.73 ± 0.67^§^	-0.130 ± 0.067	-0.714 ± 0.672	0.066 ± 0.029^§^	0.056 ± 0.070
**Brown Fat Weight (g)**	R^2^	0.008	0.006	0.087	0.067	0.028	0.007	0.074	0.109
	Regression coefficient ± SE	0.091 ± 0.160	0.005 ± 0.011	13.79 ± 7.08	14.89 ± 8.77	-0.927 ± 0.870	-4.46 ± 8.49	0.666 ± 0.374	1.85 ± 0.83^§^
**Gastrocnemius Muscle Weight (g)**	R^2^	0.005	0.005	0.021	0.029	0.029	0.011	0.015	0.001
	Regression coefficient ± SE	-0.042 ± 0.092	0.003 ± 0.006	3.95 ± 4.29	5.74 ± 5.23	-0.560 ± 0.508	-3.37 ± 4.95	0.178 ± 0.225	-0.091 ± 0.517
**Fat-Free Mass (g)**	R^2^	0.207	0.147	0.027	0.115	0.0004	0.009	0.146	0.188
	Regression coefficient ± SE	0.012 ± 0.004*	0.001 ± 0.0002^§^	0.191 ± 0.182	0.485 ± 0.213^§^	0.003 ± 0.022	0.130 ± 0.211	0.023 ± 0.009^§^	0.060 ± 0.020*
**Fat Mass (g)**	R^2^	0.009	0.188	0.074	0.160	0.062	0.009	0.155	0.028
	Regression coefficient ± SE	0.001 ± 0.002	0.0003 ± 0.0001*	0.138 ± 0.077	0.248 ± 0.090*	-0.015 ± 0.009	-0.054 ± 0.092	0.010 ± 0.004*	0.010 ± 0.009

First row of numbers for each model shows how well the data fits the fitted regression line, *R*^2^, for the equation. Subsequent rows of numbers for each model show the regression coefficient, or slope of the fitted line, ± SE. BMC, bone mineral content; BMD, bone mineral density.

^§^p < 0.05;

*p < 0.01.

The summary of the regression analyses is consistent with the bone phenotype seen in the results of the biomechanical performance testing and the PCA, where the high-fat diets increase the strength of the bone, but makes it more brittle. The regression analysis gives insight into which indices of fat and fat-free mass have the greater impact on markers of bone status, including size, strength, and brittleness of the bone. Both fat-free mass and fat-mass are good predictors of bone size and mineralization, shown by a strong correlation between increased fat-free mass with increased cross-sectional area and BMD. The best marker to predict bone strength, defined as maximum load a bone can tolerate before fracture, is total fat mass, including both visceral and subcutaneous fat weights. The brittleness of the bone is most strongly predicted by subcutaneous fat and visceral fat weights, which as their weight increases, the brittleness of the bone, measured as post-yield displacement, increases as well.

### High-Fat Diets Did Not Lead to Inflammation

When spleen mass was expressed relative to body weight, there were no differences amongst the four diets: 60%-fat casein, 0.331 ± 0.016 g/100g body weight; 60%-fat GMP, 0.345 ± 0.026 g/100g body weight; 13%-fat casein, 0.380 ± 0.025 g/100g body weight; and 13%-fat GMP, 0.398 ± 0.020 g/100g body weight. Concentrations of pro-inflammatory cytokines, such as interferon-γ and tumor necrosis factor-α, were measured in the plasma and, consistent with the results of spleen mass, there were no significant differences seen between mice fed the four diets, S2 Table.

## Discussion

We demonstrate differential effects on fatty acid oxidation and bone phenotype in female mice fed diets differing in fat content and protein source. Female mice with high-fat diet induced obesity show increased oxidative capacity of the gastrocnemius muscle and brown adipose tissue and stronger bones that are more brittle compared with feeding a 13% fat control diet. Feeding GMP as the main protein source in a high-fat diet did not alter body composition or oxidative capacity of muscle tissue, although oxidative capacity of subcutaneous fat was increased. A 13%-fat GMP diet increased total body fat-free mass and mineralization and increased femoral length while reducing brittleness of the femora compared with a 13%-fat casein diet.

### Female Mice Fed High-Fat Diets Show Increased Fat Oxidation Capacity Without Insulin Resistance or Inflammation

There were significant increases in specific activity of ex vivo fatty acid oxidation, indicating a greater capacity for fat oxidation, in both the gastrocnemius muscle and brown fat of female mice fed the 60% fat diets compared to female mice fed the control diets. Uptake of the extra fatty acids into muscle is likely the primary adaptation to the high-fat diet as muscle has greater mass than brown fat and we did not see an increase in UCP-1 expression in brown fat. This is consistent with evidence that male rodents can adapt to excess lipid accumulation by increasing uptake and utilization of fatty acids into skeletal muscle [41–43]. There was little evidence of impaired glucose metabolism or inflammation in the mice fed the high-fat diets compared to mice fed control diets, suggesting that female mice, unlike male mice [38] adapt to chronic ingestion of a high-fat diet. This differential gender response is likely due to the protective effects of estrogen, as others have shown that female mice who are fed a high-fat diet have reduced fat mass associated with reduction in metabolic impairments, including reduced insulin resistance, compared with female mice, who are ovariectomized or unable to synthesize estrogen [44, 45]. When ovariectomized mice are treated with estradiol, to mimic estrogen replacement therapy in postmenopausal women, it improves both hepatic and muscle insulin sensitivity suggesting that estrogen is functioning to prevent insulin resistance [46]. Obesity is associated with a chronic, low-grade inflammation, which also contributes to the development of insulin resistance [47]. When male and female mice are compared in their response to a high-fat diet both gained similar weight, but the female mice did not develop low-grade systemic inflammation. This is likely due to the fact that the female mice were able to expand populations of anti-inflammatory regulatory T cells in visceral adipose tissue [48]. This protection of estrogen against metabolic dysfunctions associated with high-fat diet induced obesity may explain why we did not see development of insulin resistance or systemic inflammation in our female mice.

### Female Mice Fed High-Fat Diets Have Strong Yet Brittle Bones

We investigated the biomechanical performance properties of femora from female mice fed a high-fat diet. When male mice are fed a high-fat diet, a bone phenotype is displayed where the size, or cross-sectional area, of the bone increases, but the size-independent properties of bone ductility decreases [28]. This suggests that the bone improves enough to match the size and load demands of the excess weight and no excess energy is expended to further improve bone quality [49]. The most striking difference between male and female mice in response to the high-fat diet, is that in female mice the yield and maximum loads tolerated before fracture increased in our study; whereas, in male mice, no difference was seen between chow and high-fat diets in regards to yield and max loads [28]. Thus, the primary difference is that bones get stronger with high-fat feeding in female mice, but not male mice. The impact of estrogen is likely why female mice have a different bone phenotype, i.e. become stronger, than male mice in response to high-fat feeding [50]. When female mice are given an ovariectomy, they develop an osteoporotic phenotype resulting from greater bone turnover where resorption is greater than deposition of the bone [51, 52]. Likewise, male mice with high-fat induced obesity had greater bone loss in the cancellous bone compartment compared with female mice [53].

Another hormone that impacts bone in relation to fat is leptin. Leptin is secreted by adipocytes and is present in increased concentrations in obesity, consistent with what we report in our female mice with high-fat diet induced obesity. The impact of increased leptin concentrations on bone, whether it is beneficial or antagonistic, is under debate [28, 54]. The leptin receptor is present on osteoblasts, the bone building cells, and thus, the presence of leptin increases proliferation and differentiation of osteoblasts [55], which may lead to the increase in size and strength as we report. Leptin also regulates osteoclasts, which functions to resorb bone, by increasing concentrations of receptor activator of nuclear transcription factor κB ligand, which differentiates preosteoclasts into osteoclasts and increases bone resorption [56, 57]. An increase in bone resorption, potentially mediated by increased leptin levels, may explain why we see increased brittleness in the femora of our female mice.

### Markers of Body Composition, Including Fat and Fat-Free Mass, Correlate with Markers of Bone Status

The link between body composition and bone status has been debated as traditionally a higher body mass, including those individuals who are obese, has been associated with a higher bone mass [58–60]. More recent work, however, suggests that fat mass either is not associated or is negatively related to bone mass [61, 62]. We investigated the relationship between body composition and bone status by examining correlations between measures of body fat, fat-free mass, and mineral content and markers of bone size and biomechanical performance. Our data is consistent with previous reports, that the increased loading of the bone due to greater body mass, increases size or cross-sectional area of the bone, but impairs the quality of the bone as the ductility of the bone is decreased [26]. A unique aspect to our study is that we were able to correlate body composition with biomechanical performance data and found that all markers of body fat content were negatively associated with post-yield displacement or ductility of the bone. This suggests that it is the excess body fat that is causing the bones of female mice to become brittle. Having a bone that is brittle is of clinical concern as it will bend very little before snapping like a piece of chalk, whereas a ductile bone will bend quite a bit before it undergoes a complete fracture.

### GMP Modulates Body Composition

We previously demonstrated that GMP reduces body fat in female mice, compared to a 17% fat kcal casein diet, concurrent with its ability to increase whole body fat oxidation based on a lower respiratory exchange ratio [17]. This gave reason to determine if greater fat oxidation capacity was a mechanism by which GMP functions to reduce body fat. Fat oxidation in liver, heart, gastrocnemius muscle, and visceral fat, were not altered with GMP feeding. However, there was a significant 3-fold increase in the rate of fat oxidation in subcutaneous fat, and a trend for greater fat oxidation in brown fat in mice fed a high-fat GMP diet relative to mice fed the high-fat casein diet. These results are consistent given that subcutaneous fat may contain a unique cell type, called beige cells that function similar to brown fat [63]. We conclude that an increase in oxidative capacity of muscle tissues is not the primary mechanism by which GMP reduces body fat. Another mechanism we explored was to examine relative rates of expression of the mitochondrial genes that are involved in the regulation of fatty acid oxidation, including PPAR-α, CPT1-α, CPT1-β, PGC1-α, and PPAR-γ [64]. Interestingly, a reduction in relative expression of PPAR-α mRNA is seen in visceral fat in mice fed the GMP diets, which suggests that, in visceral fat tissue, GMP is not upregulating fatty acid oxidation, as PPAR-α is known to increase β-oxidation in the mitochondria as well as upregulate other genes involved in fatty acid oxidation [65]. There were no differences in expression of the other genes as well suggesting that GMP is not altering transcriptional regulation of fatty acid oxidation.

GMP modulated body composition and energy balance in the current study, although body fat content was not reduced as previously reported [16–18]. Mice fed the high-fat GMP diet, while having the same total caloric intake as mice fed the high-fat casein diet, had a significantly lower body weight at the end of the study, and mice fed 13%-fat GMP had a greater total caloric intake than mice fed 13%-fat casein yet they had equal body weights at the end of the study. This is suggestive that GMP is altering energy intake and metabolism to reduce fat accumulation in both high-fat and control diets. Increased levels of metabolites derived from threonine metabolism may impact energy metabolism via anapleurotic effects on the TCA cycle; this deserves further research. We found that GMP, in both the high-fat and control diets, significantly reduced plasma concentrations of ghrelin compared to the casein diets, which can function to reduce hunger and increase satiety. Individuals with PKU who were fed a breakfast containing GMP also had reduced levels of ghrelin [10]. While plasma ghrelin was significantly reduced in our mice, we did not see a correlating reduction in food intake, as food intake was measured over the entire study and plasma ghrelin was measured in fasting conditions at the end of the study.

### GMP Improves Bone Status in Control Diets

We previously demonstrated that GMP can improve bone size and biomechanical properties relative to other protein sources, including casein [22]. In our mice fed the GMP diets, we saw significant increases in total body BMC, femoral bone perimeter, and femoral bone length. This may reflect GMP’s influence to improve calcium balance as other investigators have demonstrated that GMP can increase calcium absorption, particularly when calcium availability is low, [66] and also decrease urinary calcium excretion [67].

The prebiotic properties of GMP including increased cecal short-chain fatty acids (SCFA) may also explain the positive effects of GMP on bone [5]. Previous studies have shown that SCFAs, in particular butyrate, can affect epigenetic regulation of chromatin structure leading to changes in gene expression that may be involved with mineral absorption [68–70]. A recent study linking prebiotic fibers with modulation of the microbiota showed decreased cecal pH and an increase in bifidobacteria that were associated with greater absorption and retention of calcium and magnesium, increased femur and tibia breaking strength, and increased femur BMD [71].

The skeleton is a source of both mesenchymal and hematopoietic stem cells, which give rise to adipocytes and osteoblasts as well as osteoclasts and T-cells, respectively [72]. Modulation of the microbiota impacts the inflammatory state of an individual, which can alter production of cytokines associated with the differentiation of osteoblasts and osteoclasts. Germ-free mice showed an increase in bone mass with fewer osteoclasts per bone surface, and when these mice were colonized, bone mass was normalized in association with a greater number of T-cells [72]. The anti-inflammatory properties that have been associated with GMP [5, 17] may be linked to improving bone mass in the mice by altering the differentiation of mesenchymal and hematopoietic stem cells.

In summary, female mice fed the high-fat diet showed increased strength of femora in biomechanical performance testing, but these bones were more brittle than the femora of mice that were fed a control diet. Estrogen in female mice likely accounts for greater bone strength in female mice with high-fat diet induced obesity, because male mice with high-fat diet induced obesity do not show stronger bones. Further research is needed to understand how gender and diet-induced adiposity modulates bone quantity and quality.

## Supporting Information

S1 Fig Insulin tolerance test. Insulin tolerance tests were performed on female mice fed high-fat casein, high-fat GMP, low-fat casein, or low-fat GMP diets. Insulin was administered by intraperitoneal injection after a 4 hour fast. The data are expressed as means ± SE. Nos. in parentheses indicate sample size. *, p<0.05.
(TIF)

S1 Table Plasma hormone concentrations in female mice fed a 60%-fat casein, 60%-fat GMP, 13%-fat casein, or 13%-fat GMP diet. (DOCX)

S2 Table Plasma cytokine concentrations in female mice fed a 60%-fat casein, 60%-fat GMP, 13%-fat casein, or 13%-fat GMP diet. (DOCX)
