# Supplementary figures and images for: Differential Effects of Dietary Fat Content and Protein Source on Bone Phenotype and Fatty Acid Oxidation in Female C57Bl/6 Mice
Source: PLoS One. 2016 Oct 3;11(10):e0163234. doi: 10.1371/journal.pone.0163234 (PMC5047596; doi:10.1371/journal.pone.0163234)

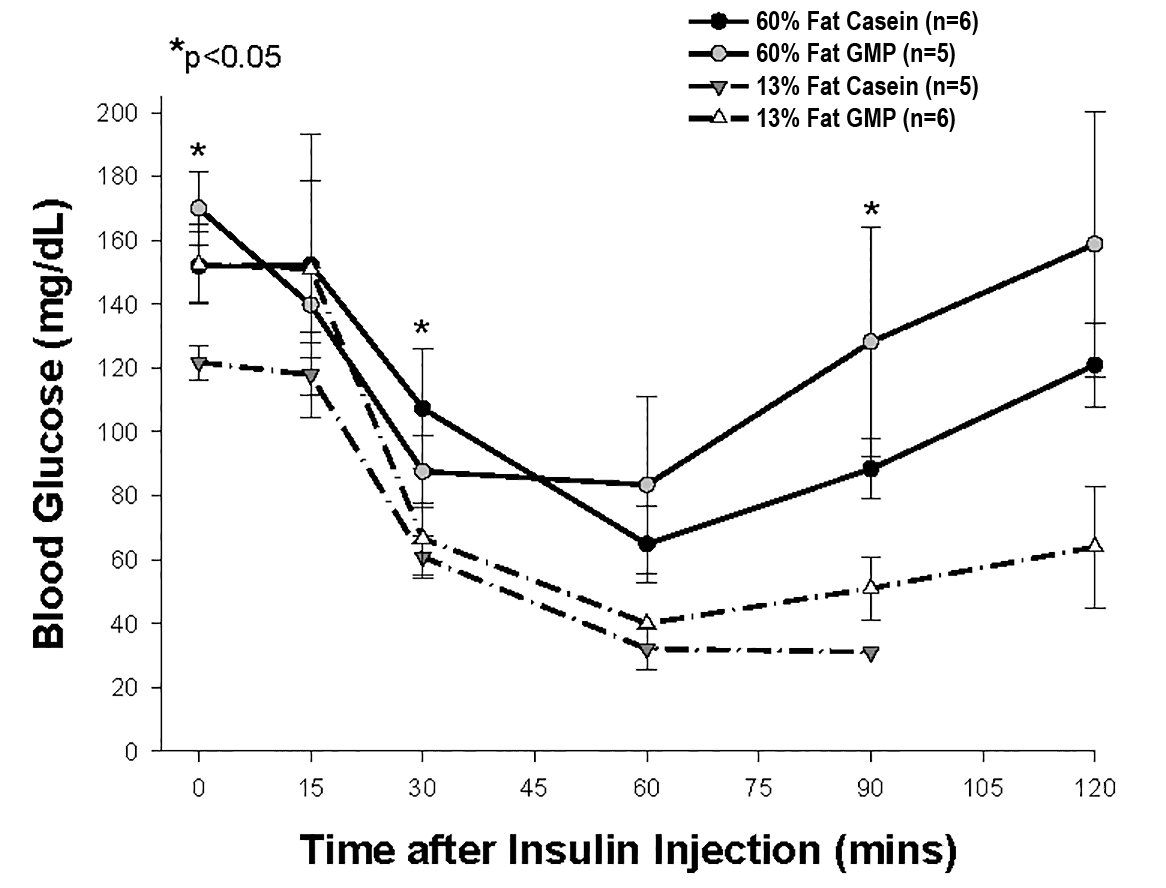

Supplement: S1 Fig — Insulin tolerance tests were performed on female mice fed high-fat casein, high-fat GMP, low-fat casein, or low-fat GMP diets. Insulin was administered by intraperitoneal injection after a 4 hour fast. The data are expressed as means ± SE. Nos. in parentheses indicate sample size. *, p<0.05. (TIF) [file pone.0163234.s001.tif]
