# Supplementary material for: Differential Effects of Dietary Fat Content and Protein Source on Bone Phenotype and Fatty Acid Oxidation in Female C57Bl/6 Mice
Source: PLoS One. 2016 Oct 3;11(10):e0163234. doi: 10.1371/journal.pone.0163234 (PMC5047596; doi:10.1371/journal.pone.0163234)
Supplement: S1 Table — (DOCX) [file pone.0163234.s002.docx]

**S1 Table.** Plasma hormone concentrations in female mice fed either a 60%-fat casein, 60%-fat GMP, 13%-fat casein, or 13%-fat GMP diet.

|  | 60%-fat |  | 13%-fat |  |
| --- | --- | --- | --- | --- |
| Hormone | **Casein** | **GMP** | **Casein** | **GMP** |
| *N* | 6 | 9 | 10 | 10 |
| GIP (pg/mL) | 284 ± 63 | 355 ± 33 | 316 ± 26 | 245 ± 31 |
| GLP-1 (pg/mL) | 63.0 ± 15.7 | 75.5 ± 8.9 | 61.8 ± 8.4 | 61.1 ± 11.4 |
| PAI-1 (pg/mL) | 616 ± 96 | 927 ± 108 | 735 ± 69 | 650 ± 76 |
| Resistin (ng/mL) | 192 ± 114 | 219 ± 65 | 275 ± 74 | 172 ± 38 |
| Glucagon (pg/mL) | 241 ± 42 | 319 ± 74 | 320 ± 43 | 294 ± 39 |

Values are means ± SE of raw data; *N*, no of mice; GIP, gastric inhibitory polypeptide; GLP-1, Glucagon-like peptide-1; PAI-1, Plasminogen activator inhibitor-1. ^a^ protein effect, ^b^ fat effect, ^c^ protein*fat effect.
