# Supplementary material for: Differential Effects of Dietary Fat Content and Protein Source on Bone Phenotype and Fatty Acid Oxidation in Female C57Bl/6 Mice
Source: PLoS One. 2016 Oct 3;11(10):e0163234. doi: 10.1371/journal.pone.0163234 (PMC5047596; doi:10.1371/journal.pone.0163234)
Supplement: S2 Table — (DOCX) [file pone.0163234.s003.docx]

**S2 Table.** Plasma cytokine concentrations in female mice fed either a 60%-fat casein, 60%-fat GMP, 13%-fat casein, or 13%-fat GMP diet.

|  | 60%-fat |  | 13%-fat |  |
| --- | --- | --- | --- | --- |
| Cytokine | **Casein** | **GMP** | **Casein** | **GMP** |
| *N* | 6 | 8 | 10 | 10 |
| IL-1a (pg/mL) | 7.56 ± 0.44 | 9.39 ± 1.20 | 11.56 ± 2.19 | 8.84 ± 0.72 |
| IL-1b (pg/mL) | 229 ± 27 | 299 ± 43 | 276 ± 29 | 257 ± 28 |
| IL-3 (pg/mL)^b^ | 11.5 ± 2.2 | 7.4 ± 1.5 | 5.6 ± 1.0 | 5.9 ± 0.5 |
| IL-4 (pg/mL) | 11.2 ± 0.9 | 12.3 ± 1.0 | 12.9 ± 1.4 | 11.9 ± 0.7 |
| IL-5 (pg/mL) | 3.3 ± 2.5 | 23.7 ± 5.5 | 14.6 ± 2.5 | 13.6 ± 1.7 |
| IL-6 (pg/mL) | 5.61 ± 1.36 | 6.98 ± 1.37 | 7.74 ± 1.11 | 5.90 ± 0.67 |
| IL-10 (pg/mL) | 55.0 ± 6.4 | 80.2 ± 13.6 | 81.6 ± 7.1 | 76.2 ± 9.4 |
| IL-12 (p40) (pg/mL) | 166 ± 37 | 157 ± 16 | 151 ± 6 | 121 ± 19 |
| IL-12 (p70) (pg/mL) | 138 ± 23 | 194 ± 9 | 184 ± 18 | 180 ± 21 |
| IL-13 (pg/mL) | 181 ± 24 | 197 ± 15 | 233 ± 25 | 227 ± 15 |
| IL-17a (pg/mL) | 55.5 ± 12.9 | 70.5 ± 9.3 | 82.9 ± 20.2 | 56.3 ± 10.0 |
| Eotaxin (pg/mL) | 580 ± 180 | 616 ± 176 | 1094 ± 172 | 748 ± 116 |
| G-CSF (pg/mL)^c^ | 30.9 ± 5.2 | 43.8 ± 6.6 | 46.1 ± 3.9 | 34.1 ± 3.5 |
| GM-CSF (pg/mL) | 44.7 ± 8.3 | 77.9 ± 26.8 | 57.2 ± 5.6 | 53.0 ± 7.0 |
| IFN-γ (pg/mL) | 28.7 ± 4.9 | 27.5 ± 2.6 | 36.5 ± 5.0 | 31.2 ± 3.1 |
| KC (pg/mL) | 22.9 ± 4.1 | 28.7 ± 3.5 | 30.7 ± 3.2 | 22.3 ± 2.4 |
| MCP-1 (pg/mL)^b^ | 178 ± 22 | 256 ± 33 | 272 ± 27 | 272 ± 23 |
| MIP-1a (pg/mL) | 8.7 ± 1.5 | 11.2 ± 1.7 | 10.7 ± 1.6 | 10.7 ± 1.1 |
| MIP-1b (pg/mL) | 33.6 ± 4.9 | 39.7 ± 3.5 | 43.0 ± 4.9 | 39.7 ± 2.5 |
| RANTES (pg/mL) | 7.7 ± 0.9 | 14.2 ± 2.4 | 11.9 ± 2.1 | 14.4 ± 2.2 |
| TNF-α (pg/mL) | 295 ± 36 | 363 ± 28 | 357 ± 35 | 329 ± 28 |

Values are means ± SE of raw data; *N*, no of mice; IL, interleukin; G-CSF, Granulocyte-colony stimulating factor; GM-CSF, Granulocyte-macrophage colony stimulating factor; IFN, interferon; KC, keratinocyte chemoattractant; MCP, monocyte chemoattractant protein; MIP, macrophage inflammatory protein; RANTES, regulated on activation, normal T cell expressed and secreted; TNF, tumor necrosis factor. ^a^ protein effect, ^b^ fat effect, ^c^ prot*fat effect. **IL-3:** High-fat fed mice had greater IL-3 than control diet fed mice. **G-CSF:** Mice fed the 13%-fat casein diet had significantly greater G-CSF than mice fed the 60%-fat casein diet. **MCP-1:** Control diet fed mice had greater plasma MCP-1 than mice fed the high-fat diets.
